# Aberrant Water Homeostasis Detected by Stable Isotope Analysis

**DOI:** 10.1371/journal.pone.0011699

**Published:** 2010-07-21

**Authors:** Shannon P. O'Grady, Adam R. Wende, Christopher H. Remien, Luciano O. Valenzuela, Lindsey E. Enright, Lesley A. Chesson, E. Dale Abel, Thure E. Cerling, James R. Ehleringer

**Affiliations:** 1 Department of Biology, University of Utah, Salt Lake City, Utah, United States of America; 2 Division of Endocrinology, Metabolism, and Diabetes, Salt Lake City, Utah, United States of America; 3 Department of Mathematics, University of Utah, Salt Lake City, Utah, United States of America; 4 IsoForensics, Inc., Salt Lake City, Utah, United States of America; 5 Department of Geology and Geophysics, University of Utah, Salt Lake City, Utah, United States of America; Mayo Clinic College of Medicine, United States of America

## Abstract

While isotopes are frequently used as tracers in investigations of disease physiology (i.e., ^14^C labeled glucose), few studies have examined the impact that disease, and disease-related alterations in metabolism, may have on stable isotope ratios at natural abundance levels. The isotopic composition of body water is heavily influenced by water metabolism and dietary patterns and may provide a platform for disease detection. By utilizing a model of streptozotocin (STZ)-induced diabetes as an index case of aberrant water homeostasis, we demonstrate that untreated diabetes mellitus results in distinct combinations, or signatures, of the hydrogen (δ^2^H) and oxygen (δ^18^O) isotope ratios in body water. Additionally, we show that the δ^2^H and δ^18^O values of body water are correlated with increased water flux, suggesting altered blood osmolality, due to hyperglycemia, as the mechanism behind this correlation. Further, we present a mathematical model describing the impact of water flux on the isotopic composition of body water and compare model predicted values with actual values. These data highlight the importance of factors such as water flux and energy expenditure on predictive models of body water and additionally provide a framework for using naturally occurring stable isotope ratios to monitor diseases that impact water homeostasis.

## Introduction

**Figure 1:**
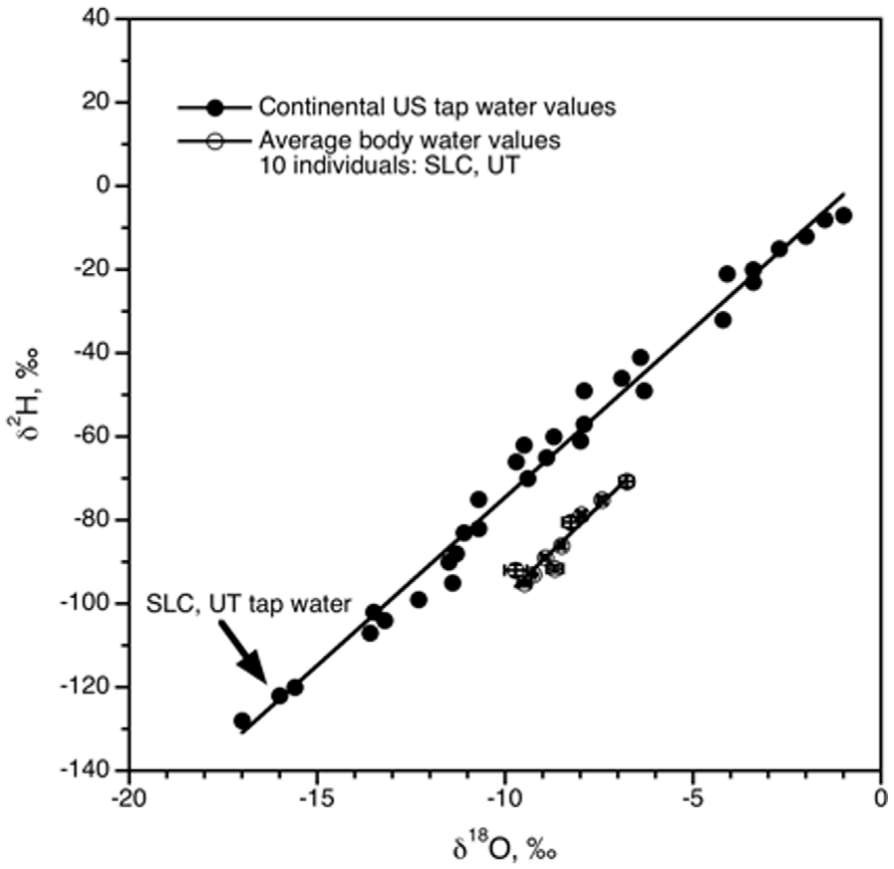
Isotopic values of continental tap water relative to body water. Measured δ^2^H and δ^18^O values of tap water samples collected from 18 states across the continental United States (•) and body water (urine) samples collected within Salt Lake City, Utah (○). The simple linear regression between the hydrogen and oxygen of tap water (δ^2^H = 8.06^18^O+6.15, r^2^ = 0.992) and body water (δ^2^H = 8.91δ^18^O−9.72, r^2^ = 0.962) are also shown.

The stable hydrogen (δ^2^H) and oxygen (δ^18^O) isotopes of body water have been used as tracers of movement and migration in the fields of anthropology [1], [2], ecology [3], [4], and forensics [5]. This is possible because the isotopic composition of body water (δ*_bw_*) is positively correlated with the isotopic composition of local precipitation (*e.g.* drinking water; δ*_dw_*) [6]–[8] and the isotopic composition of meteoric water varies geographically [9], [10]. The isotopic signature of body water is a composite of quantitative and qualitative influx and efflux factors, such as fluid and food intake, urine output, and sweat production [6], [7], [11], [12]. Although drinking water is the major contributing factor to the body water pool, the oxidation of hydrogen and oxygen atoms contained in food and water elevate the isotopic composition of body water above that of drinking water (Fig. 1). In addition to the impact that the δ^2^H and δ^18^O values of drinking water can have on the isotopic signature of body water, the volume of water processed by the body may also be a major determinant of body water signature.

The combined volume of influx and efflux factors impacting the body water pool is defined as total water flux (TWF). In healthy humans drinking water, food water, and atmospheric oxygen are the primary input factors, contributing 43%, 30%, and 20% to the body water pool, respectively. Liquid water in the form of urine and sweat are the primary outputs, constituting ∼63% of total water loss (Podlesak et al., in review). All of these factors result in an offset between the isotopic composition of the major drinking water input and the isotopic composition of body water (Fig. 1). While relatively few studies have measured TWF in humans [13], studies examining body water from animals known to have high TWF, such as dairy cows and hippopotamids [14], [15], suggest that as TWF increases, the isotopic contribution of drinking water also increases, and the δ^2^H and δ^18^O of body water approach that of drinking water.

Untreated diabetes mellitus is a state of elevated water flux. Glycosuria and polyuria ensue when blood glucose levels exceed the renal threshold [16]. Eventually, untreated diabetes results in dehydration, thirst, and increased fluid intake. We hypothesized that increased water flux in animals with untreated diabetes mellitus would yield a body water isotope signature more similar to drinking water isotope values than control animals. To test this hypothesis, we measured water intake, urine output, body water stable isotope ratios, and blood glucose values utilizing a model of streptozotocin (STZ)-induced diabetes mellitus. Treatment with streptozotocin destroys pancreatic islet cells, thus inducing insulin-dependent diabetes mellitus [17]. Additionally, we applied an isotope mass balance model to estimate the δ^2^H and δ^18^O values of mouse body water for different levels of TWF.

## Results

### TWF is higher in diabetic STZ mice relative to vehicle-treated (VEH) controls

**Figure 2:**
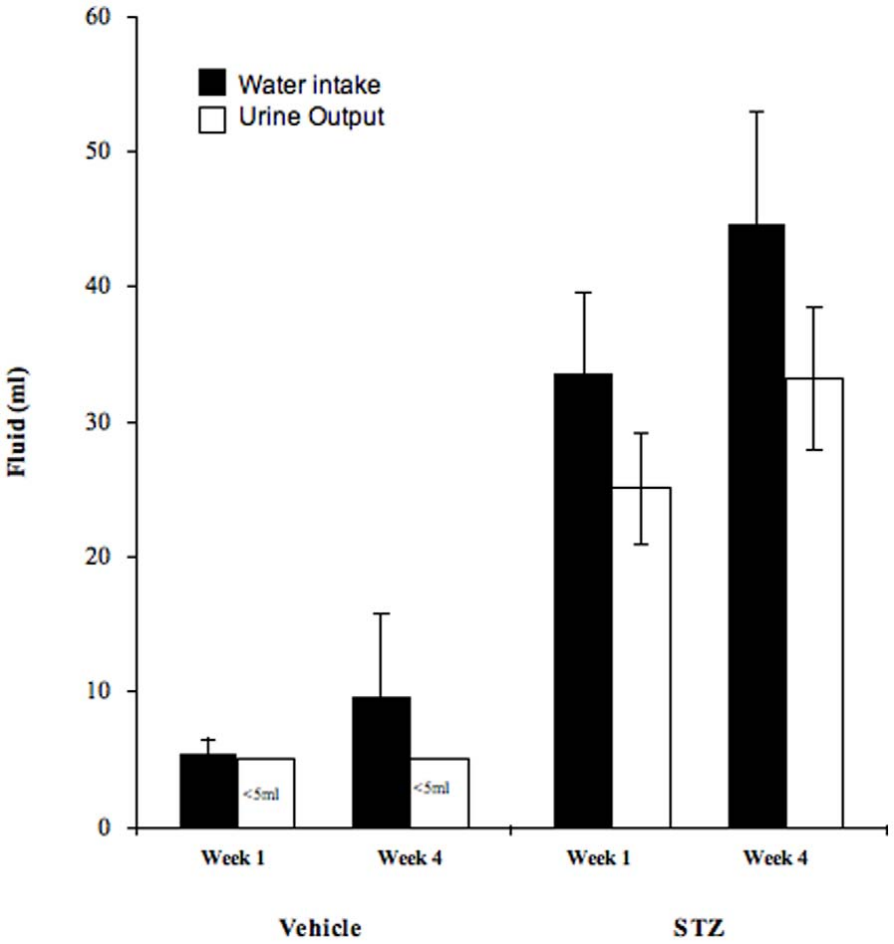
Water flux of VEH and STZ mice. Average water intake (▪) and urine output (□) from VEH treated (n = 6) and STZ treated mice (n = 6), at 1 week and 4 weeks post-injection. Bars delineate standard deviation within each group. Urine output from VEH treated mice was less than 5ml/day and below measurement precision.

Water intake of STZ mice (week 1: 33.6±6.0 ml s.d.; week 4: 44.6±8.4 ml s.d.) was five to six times that of VEH treated mice (week 1: 5.5±1.1 ml s.d.; week 4: 9.7±6.0 ml s.d.), at 1 and 4 weeks post-injection (two tailed t-test: α = 0.05, p<0.0001 and p<0.0001, respectively; Fig. 2). Additionally, average daily urine output was greater in STZ mice (week 1: 25±4.1 ml s.d.; week 4: 33.2±5.2 ml s.d.) relative to VEH treated mice (<5ml) at both week 1 and 4.

### Varying only TWF, model accurately predicts body water isotope composition

**Figure 3:**
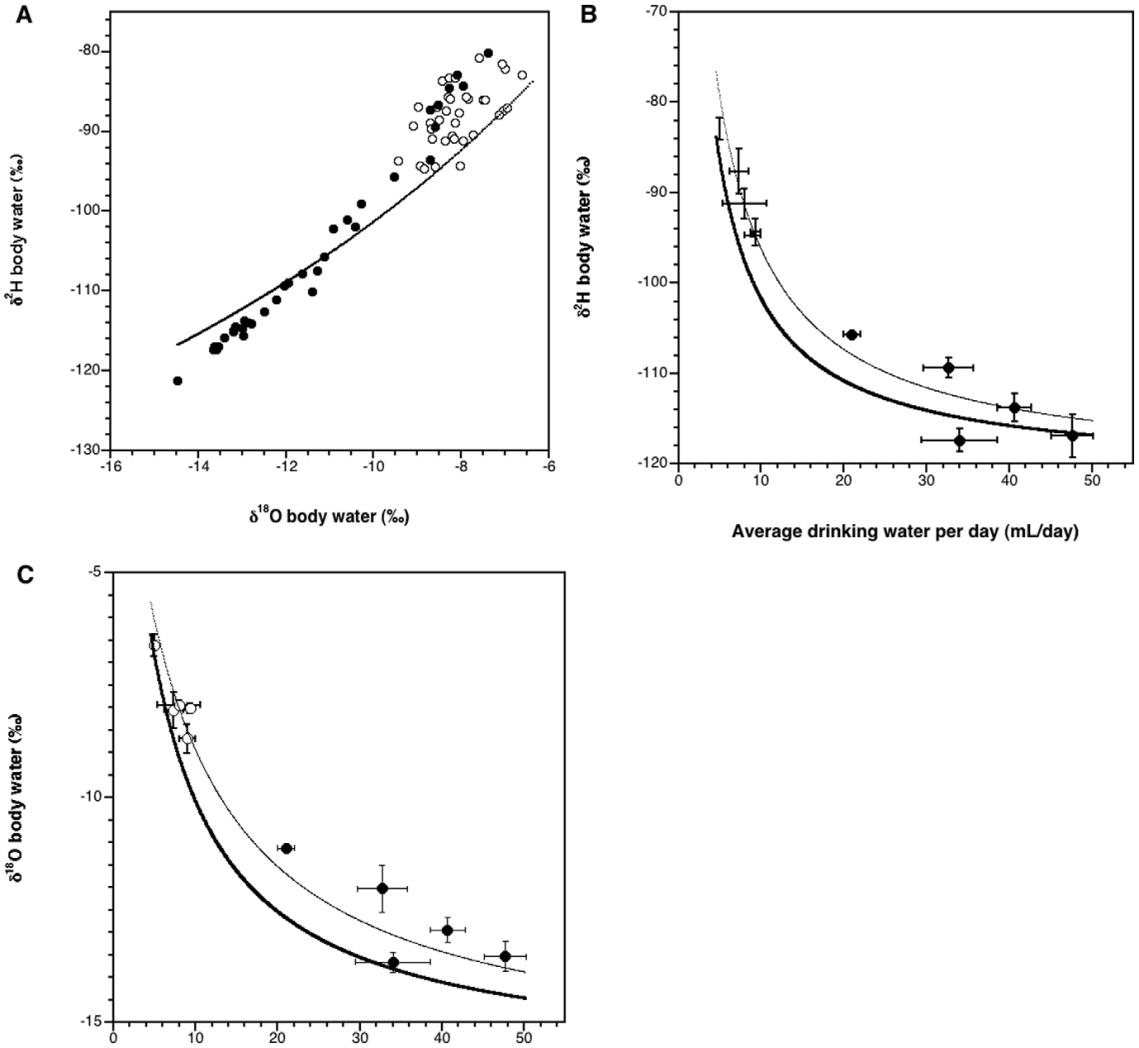
Isotopic values of body water relative to model predictions. Isotopic signature of body water of VEH (○) and STZ (•) mice relative to body water model predictions in which the only model variant is water influx. (A) Measured δ^2^H relative to δ^18^O of body water for all animals over the entire 5-week study period. The curve depicts model predictions for δ^2^H and δ^18^O values over a range of water influx values. (B) Measured δ^2^H values of body water and (C) δ^18^O values of body water relative to average drinking water per day of 9 individual animals (mL/day). The bold curve on each panel indicates values predicted by our mathematical model of the isotopic composition of body water using a total energy expenditure (TEE) of 37.4 KJ/day. Correlations between the model and measured δ^2^H and δ^18^O of body water yielded an r^2^ = 0.83 and r^2^ = 0.79, respectively. The lighter curve on each panel indicates values predicted by our mathematical model of the isotopic composition of body water using a TEE of 65.0 KJ/day. Correlations between the model and measured δ^2^H and δ^18^O of body water yielded an r^2^ = 0.96 and r^2^ = 0.96, respectively. Bars delineate standard deviation for sample analyses.

The δ^2^H and δ^18^O values of body water from STZ and VEH animals exhibited significant covariance (r^2^ = 0.99, p<0.0001 and r^2^ = 0.31, p<0.0001, respectively; Fig. 3a). Varying only the modeled influx of drinking water, the model accurately predicts both the actual range and concavity of body water δ^2^H and δ^18^O values in STZ and VEH mice as average drinking water intake increases (r^2^ = 0.83 and r^2^ = 0.79, respectively; Fig. 3b and c). The accuracy of model predictions increases for both δ^2^H and δ^18^O in STZ and VEH mice if the total energy expenditure (TEE) of animals is increased from 37.4 KJ/day to 65.0 KJ/day (r^2^ = 0.96 and r^2^ = 0.96, respectively; Fig. 3b and c)

### Body water of diabetic STZ mice is isotopically distinct from that of VEH mice

**Figure 4:**
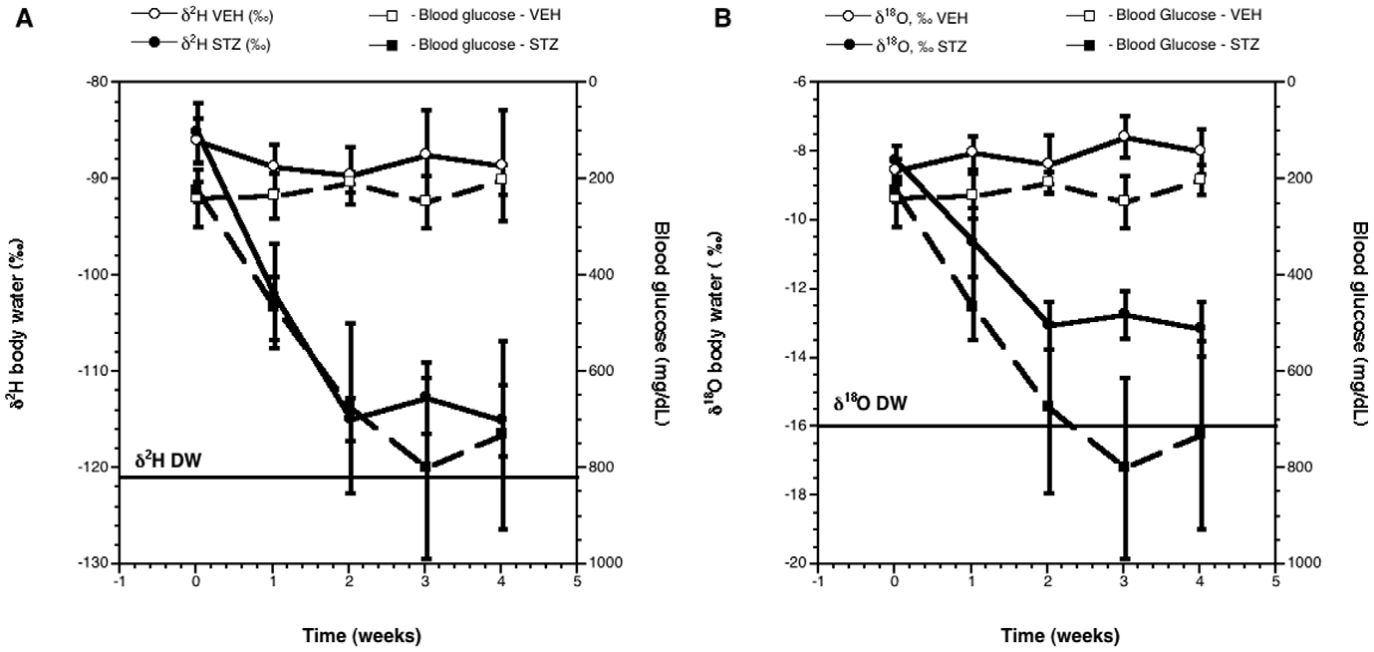
Isotopic values of body water relative to blood glucose. Isotopic signature of body water (y1 axis) and blood glucose values (y2 axis) over the 5 week study period relative to the isotopic signature of drinking water (−DW). Time 0 is prior to STZ treatment. (A) δ^2^H of body water and blood glucose values of VEH treated (○ and □, respectively; n = 7) and STZ treated (• and ▪, respectively; n = 6) mice. (B) δ^18^O of body water and blood glucose values of VEH treated (○ and □, respectively ; n = 7) and STZ treated (• and ▪, respectively; n = 6) mice. Bars delineate standard deviation for duplicate sample analyses.

There was no significant difference between STZ and VEH animals in the isotopic composition of body water or blood glucose readings at the start of the study (week 0, Fig 4). Additionally, neither the isotopic composition of body water or blood glucose of VEH animals changed significantly during the course of the study (Fig. 4). By week 1, relative to VEH animals, STZ animals had significantly lower δ^2^H and δ^18^O body water values (ANOVA: α = 0.05, p<0.0001; −88.6±2.3‰ s.d. vs. −101.9±5.4‰ s.d. and −7.9±.5‰ s.d. vs. −10.6±1.0‰ s.d., respectively; Fig. 4) and significantly higher blood glucose (ANOVA: α = 0.05, p<0.0001; 232±47 mg/dL s.d. vs. 465±66 mg/dL s.d., respectively; Fig. 4). The average isotopic composition of body water of STZ animals was significantly different at week 2 relative to week 1 (ANOVA: α = 0.05, p<0.0001; week 2: δ^2^H = −114.8±2.2‰ s.d., δ^18^O = −13±.7‰ s.d.), but there was no difference in the isotopic composition of body water of STZ animals between weeks 2, 3, and 4 (Fig. 4). This was also true for blood glucose values of STZ animals, with week 2 values significantly increased relative to week 1 (ANOVA: α = 0.05, p<0.0001; week 2: 680.9±141 mg/dL s.d.), but no difference between weeks 2, 3, and 4 (Fig. 4).

### Body water of STZ mice approaches isotopic signature of drinking water

By week 4, the δ^2^H and δ^18^O values of body water from STZ animals were similar to those of drinking water (δ^2^H = −121‰, δ^18^O = −16‰), with approximately a 6‰ and 3‰ difference between body water and drinking water H and O isotope ratios, respectively (Fig. 4). In contrast, relative to drinking water, the δ^2^H and δ^18^O values of body water from VEH animals were elevated by approximately 34‰ and 8‰, respectively (Fig. 4).

## Discussion

Water intake and urine output data (Fig. 2) indicate that TWF is higher in diabetic STZ mice relative to VEH treated controls. Consistent with our hypothesis, the δ^2^H and δ^18^O values of body water from diabetic STZ mice resembled the isotopic signature of drinking water much more closely than did the body water values of VEH mice (Fig. 4). Our results suggest that as TWF increases, the isotopic input of drinking water becomes a major determinant of the isotopic signature of body water. We hypothesize that altered water flux due to hyperglycemia, and not metabolism, is the mechanism underlying the similarity between the isotopic signatures of diabetic STZ body water and drinking water. Further, supporting the impact of water flux on the isotopic composition of body water, the mathematical model used here accurately predicts the isotopic range of body water as determined by changes only in water flux, with all other model parameters held constant (Fig. 3). Both the time series data (Fig. 4) and the data generated by the body water model (Fig. 3) indicate that the isotopic composition of body water reaches a threshold level of water flux at which point the changes in the δ^2^H and δ^18^O values of body water cannot be determined. Intuitively, this threshold is isotopically indistinguishable from the isotopic value of the drinking water input (for this study, δ^2^H = −121‰ and δ^18^O = −16‰). While this study provides convincing evidence that the mechanism behind this isotopic distinction is water flux, we plan to conduct similar studies in a model of diabetes insipidus. As opposed to diabetes mellitus, which is characterized by low insulin production or resistance and can result in hyperglycemia, diabetes insipidus is a disease state caused by either the deficiency of or insensitivity to arginine vasopressin, also known as antidiuretic hormone, resulting in elevated water flux with no impact on blood glucose. Such a study would enable us to isolate the impact of elevated water flux on body water stable isotope ratios, independent of hyperglycemia.

Disease state can significantly alter both metabolism and behavior, which in turn, will directly impact total energy expenditure (TEE). Food consumption and activity levels were not measured in this study and it is possible that these factors differed between treatment groups as hyperglycemia and resulting diabetes progressed in the STZ group. Although previous research has demonstrated no difference in the metabolic rates of VEH and STZ treatment groups [18], [19], the issue that either altered food consumption or activity may impact the isotopic composition of body water should be considered. As it would be expected for a change in mass to occur if food consumption or metabolism changed significantly, we can approach this issue indirectly by examining the respective mass changes of each group throughout the progression of the study. There was no significant change in either the average mass of the VEH group (mass = 26.5±0.6g; ANOVA: α = 0.05, p = 0.52) or the STZ group (mass = 25.0±0.8g; ANOVA: α = 0.05, p = 0.24), and thus we can surmise that the changes observed in the isotopic composition of body water are likely the sole result of altered water flux due to hyperglycemia.

The model accurately predicts both the actual range and concavity of body water δ^2^H and δ^18^O values in STZ and VEH mice as average drinking water intake increases (r^2^ = 0.83 and r^2^ = 0.79, respectively; Fig. 3b and c). While all parameters were either measured directly, or estimated from directly measurable parameters, the authors acknowledge that variation in the values assigned to these parameters may alter the accuracy of model predictions. For example, in addition to values of water flux, the model is also sensitive to values assigned TEE. The value used for TEE in this exercise was 37.4 KJ/day and was calculated based on the hourly consumption of oxygen (VO_2_) by a 25-gram control mouse housed independently. This value may be an underestimate of the actual TEE of animals housed in groups (as in this study), where between animal interactions may elevate energy expenditure. To demonstrate the models' sensitivity to TEE, we nearly doubled the value of TEE used in this study (increased from 37.4 KJ/day to 65.0 KJ/day) and applied the model over the same range of water influxes used in the previous exercise. In this case, doubling the value of TEE actually increases the accuracy with which the model predicts the isotopic composition of body water (r^2^ = 0.96 for both δ^2^H and δ^18^O; Fig. 3b and c). These exercises emphasize the importance of factors such as TWF and TEE in determining the isotopic composition of body water, particularly in reconstructive or predictive applications.

## Materials and Methods

All experiments were approved by the University of Utah Institutional Animal Care and Use Committee (IACUC protocol 09-08011). Water flux measurements (n = 5/treatment) were taken 1, 3, and 4 weeks following a 5-day low dose STZ injection protocol [20]. We determined the stable hydrogen and oxygen isotope ratios of water cryogenically extracted from blood (hereafter referred to as body water) of STZ (n = 6) and VEH (n = 7) treated controls prior to STZ injections and weekly for the 4 weeks following STZ treatment. Samples were analyzed in duplicate at the University of Utah Stable Isotope Ratio Facility for Environmental Research (SIRFER; http://sirfer.net) on a ThermoFinnigan-MAT Delta Plus XL isotope ratio mass spectrometer (Bremen, Germany) with a high temperature conversion elemental analyzer (TC/EA) attached. Samples were pyrolyzed at 1400°C to produce H_2_ and CO gas. Resultant gases were separated on a 1-m, 0.25 in (outer diameter) molecular sieve 5Å gas chromatography column (Costech Analytical, Valencia, CA, USA). Water samples were introduced to the pyrolysis column using a PAL autosampler (LEAP Technologies, Carrboro, NC, USA) and analyzed alongside a set of three laboratory water reference materials previously calibrated to the Vienna Standard Mean Ocean Water (VSMOW) scale. The analytical precision for samples was ±1.55‰ and ±0.17‰ for H and O, respectively.

### Stable Isotope Notation

Stable isotope abundances are reported in *d*-notation as parts per thousands (‰), where
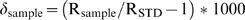
and *R*_sample_ and *R*_standard_ are the molar ratios of the rare to abundant isotope (e.g., ^2^H/^1^H) in the sample and standard, respectively. The international standard for both hydrogen and oxygen stable isotope analysis is VSMOW.

### Modeling

We applied a mechanistic model describing the isotopic composition of body water to determine if we could accurately predict the H and O isotope ratios of body water of STZ and VEH mice. Similar models have been applied to reconstruct paleoclimate from the δ^18^O value of fossil biogenic phosphate [6], [21], [22] and to determine energy expenditure via the doubly labeled water technique [11], [23].

Assuming an animal is in isotopic equilibrium, the general mass balance equation can be written as
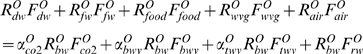
for 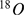, and similarly
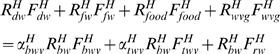
for 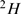. Isotope abundances are expressed as ratios (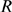) of heavy to light isotopes, fluxes (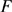) are in units mole/day, and isotopic fractionation relative to body water is expressed as 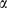. Oxygen and hydrogen influxes and effluxes considered include drinking water (*dw*), free water in food (*fw*), oxygen and hydrogen bound in food (*food*), water vapor gain (*wvg*), atmospheric oxygen (*air*), carbon dioxide (*CO_2_*), breath water vapor (*bwv*), transcutaneous water vapor (*twv*), and unfractionated remaining water loss (*rw*) which includes urine, fecal water, and sweat.

Solving for the isotope ratio of body water yields 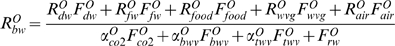 for oxygen, and 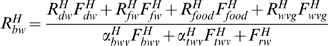 for hydrogen.

Influxes and effluxes of oxygen and hydrogen fall into two categories, those associated with free water (*dw*, *fw*, *wvg*, *bwv*, *twv*, *rw*) and those derived from the metabolism of carbohydrates, fats, and protein (*food*, *air*, *co2*). Oxidation reactions for glucose, palmitic acid, and alanine are considered first order representatives of carbohydrate, fat, and protein substrates [21]. The flux terms associated with metabolism are therefore functions of diet composition, specifically the fraction of energy derived from carbohydrates, fats, and protein, as well as total energy expenditure. All parameters were either measured directly or estimated from directly measurable parameters (Table 1). Animals were housed in a temperature controlled facility and facility-recorded averages were used for temperature (20°C) and humidity (20%) inputs in the model. Modeling exercises were performed using two values of total energy expenditure (TEE): 37.4 KJ/day and 65.0 KJ/day. For each TEE value, the drinking water influx (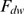) was manipulated to simulate the measured range (5–50 mL/day), while all other parameters were held constant.

**Table 1:** Values used in body water modeling.

Influx	Oxygen Flux (TEE = 37.4 KJ/day, 65.0 KJ/day)	Hydrogen Flux (TEE = 37.4 KJ/day, 65.0 KJ/day)	Source
*dw – drinking water*	0.125–1.39	0.250–2.78	measured
*fw – food water*	0	0	measured
*food – bound O and H*	0.0315, 0.0547	0.0734, 0.128	[18], [21]
*wvg – water vapor gain*	0.00602, 0.0105	0.0120, 0.0209	[21]
*air – atmospheric O*	0.0761, 0.132	n/a	[18], [21]

Efflux	Oxygen Flux (TEE = 37.4 KJ/day, 65.0 KJ/day)	Hydrogen Flux (TEE = 37.4 KJ/day, 65.0 KJ/day)	Source
*CO_2_ – carbon dioxide*	0.0709, 0.123	n/a	[18], [21]
*bwv – breath water vapor*	0.0577, 0.100	0.115, 0.201	[6]
*Twv – transcutaneous water vapor*	0.0463	0.0925	[23]
*rw – remaining water*	0.0637–1.39	0.127–2.78	calculated

Fractionation Factors	Oxygen	Hydrogen	Source
*α_co2_*	1.038	n/a	[24]
*α_bwv_*	0.991	0.946	[23], [25]
*α_twv_*	0.981	0.935	[23], [25]

Isotope Ratio	Oxygen (δ^18^O)	Hydrogen (δ^2^H)	Source
*dw – drinking water*	−16	−121	measured
*fw – food water*	n/a	n/a	n/a
*food – bound O and H*	23.9	−97.9	measured
*wvg – water vapor gain*	−25.5	−165	[6], [26]
*air – atmospheric O*	15.1	n/a	[6]

Where applicable, values for modeling exercises completed with a total energy expenditure of 37.4 and 65.0 KJ/day are included.
